# Supplementary material for: Combinatorial programming of human neuronal progenitors using magnetically-guided stoichiometric mRNA delivery
Source: eLife. 2018 May 1;7:e31922. doi: 10.7554/eLife.31922 (PMC5959718; doi:10.7554/eLife.31922)
Supplement: Supplementary file 1. [file elife-31922-supp1.docx]

**Supplementary Table 1: List and details of top and bottom magnets used in this work**
