# Supplementary material for: Combinatorial programming of human neuronal progenitors using magnetically-guided stoichiometric mRNA delivery
Source: eLife. 2018 May 1;7:e31922. doi: 10.7554/eLife.31922 (PMC5959718; doi:10.7554/eLife.31922)
Supplement: Supplementary file 2. [file elife-31922-supp2.docx]

**Supplementary Table 2: List of TaqMan qRT-PCR assays used in this research**
