# Supplementary material for: Combinatorial programming of human neuronal progenitors using magnetically-guided stoichiometric mRNA delivery
Source: eLife. 2018 May 1;7:e31922. doi: 10.7554/eLife.31922 (PMC5959718; doi:10.7554/eLife.31922)
Supplement: Transparent reporting form [file elife-31922-transrepform.docx]

***eLife’s* transparent reporting form**

We encourage authors to provide detailed information *within their submission* to facilitate the interpretation and replication of experiments. Authors can upload supporting documentation to indicate the use of appropriate reporting guidelines for health-related research (see [EQUATOR Network](http://www.equator-network.org/%20)), life science research (see the [BioSharing Information Resource](https://biosharing.org/)), or the [ARRIVE guidelines](http://www.plosbiology.org/article/info:doi/10.1371/journal.pbio.1000412) for reporting work involving animal research. Where applicable, authors should refer to any relevant reporting standards documents in this form.

If you have any questions, please consult our Journal Policies and/or contact us: [editorial@elifesciences.org](mailto:editorial@elifesciences.org).

**Sample-size estimation**

- You should state whether an appropriate sample size was computed when the study was being designed
- You should state the statistical method of sample size computation and any required assumptions
- If no explicit power analysis was used, you should describe how you decided what sample (replicate) size (number) to use

Please outline where this information can be found within the submission (e.g., sections or figure legends), or explain why this information doesn’t apply to your submission:

Sample sizes were chosen based on standards in the literature for cell culture/qPCR replicates. We have done 3 biological replicates (separate cell culture samples cultured independently) for qPCR analyses per condition.

**Replicates**

- You should report how often each experiment was performed
- You should include a definition of biological versus technical replication
- The data obtained should be provided and sufficient information should be provided to indicate the number of independent biological and/or technical replicates
- If you encountered any outliers, you should describe how these were handled
- Criteria for exclusion/inclusion of data should be clearly stated
- High-throughput sequence data should be uploaded before submission, with a private link for reviewers provided (these are available from both GEO and ArrayExpress)

Please outline where this information can be found within the submission (e.g., sections or figure legends), or explain why this information doesn’t apply to your submission:

**Co-expression analysis:**

**Fig. 2c** caption: "Both PCC and MOC represent average values from 3 independent experiments."

**Gene expression analysis (qRT-PCR):**

Methods: "Three individual samples with three replicates each were used for gene expression analysis and the data was normalized to GAPDH."

**Fig. 3d,e** caption: "All quantifications were done with n = 3 independent experiments (mean ± s.e.m), ***(p<0.001), **(p<0.01), *(p<0.05) (compared to control, Dunnett's test)."

**Fig. 4c** caption: "Quantifications were done with n=3 independent experiments (mean ± s.e.m), ***p<0.001, **p<0.01, *p<0.05 (compared to control, Student’s *t*-test)."

**Cell counting and statistical analysis:**

Methods: "For non-spotting experiments, a total of 27 random images were taken for each condition from three independent experiments. For spotting experiments, images were taken from spotted areas in three independent experiments."

**Statistical reporting**

- Statistical analysis methods should be described and justified
- Raw data should be presented in figures whenever informative to do so (typically when N per group is less than 10)
- For each experiment, you should identify the statistical tests used, exact values of N, definitions of center, methods of multiple test correction, and dispersion and precision measures (e.g., mean, median, SD, SEM, confidence intervals; and, for the major substantive results, a measure of effect size (e.g., Pearson's r, Cohen's d)
- Report exact p-values wherever possible alongside the summary statistics and 95% confidence intervals. These should be reported for all key questions and not only when the p-value is less than 0.05.

Please outline where this information can be found within the submission (e.g., sections or figure legends), or explain why this information doesn’t apply to your submission:

**Gene expression analysis (qRT-PCR):**

**Fig. 3d,e** caption: "All quantifications were done with n = 3 independent experiments (mean ± s.e.m), ***(p<0.001), **(p<0.01), *(p<0.05) (compared to control, Dunnett's test)."

**Fig. 4b,c** caption: "Quantifications were done with n=3 independent experiments (mean ± s.e.m), ***p<0.001, **p<0.01, *p<0.05 (compared to control, Student’s *t*-test).

(For large datasets, or papers with a very large number of statistical tests, you may upload a single table file with tests, Ns, etc., with reference to sections in the manuscript.)

**Group allocation**

- Indicate how samples were allocated into experimental groups (in the case of clinical studies, please specify allocation to treatment method); if randomization was used, please also state if restricted randomization was applied
- Indicate if masking was used during group allocation, data collection and/or data analysis

Please outline where this information can be found within the submission (e.g., sections or figure legends), or explain why this information doesn’t apply to your submission:

No clinical patients and/or animals were used in our experiments that required group allocation. Masking was not used during collection and/or data analysis because all experimental outcomes were assessed using quantitative metrics that were extracted automatically from raw image files and TaqMan qRT-PCR amplification curves using automated algorithms and commercially available software. This ensures that all experimental groups were processed in an unbiased manner using identical criteria/code. This includes co-expression analysis (**Fig. 2c**), gene expression analysis (**Fig. 3d,e**; **Fig. 4c**), and immunocytochemistry analysis (**Fig. 4b**).

**Additional data files (“source data”)**

- We encourage you to upload relevant additional data files, such as numerical data that are represented as a graph in a figure, or as a summary table
- Where provided, these should be in the most useful format, and they can be uploaded as “Source data” files linked to a main figure or table
- Include model definition files including the full list of parameters used
- Include code used for data analysis (e.g., R, MatLab)
- Avoid stating that data files are “available upon request”

Please indicate the figures or tables for which source data files have been provided:

We include a "code availability" subsection to the **Methods**, where we provide the entire software that controls the dual-magnet array hardware used for all the spotting experiments in the paper:

**“Code availability**

LabView files for programming of the dual-magnet array are available online from GitHub (https://github.com/rezaie99/ELIFE-050518).”

We also include the source data files, including co-transfection efficiencies (**Fig. 2**) and TH expression levels under different transcription factor combinations (delivered either during proliferative or post-induction stages) (**Fig 3**).

A "Key Resources Table" that lists relevant catalog numbers, database IDs, RRIDs, and public/commercial sources for the various genes, antibodies, cell lines, and primers is included at the beginning of the **Materials and Methods** section.
